# Y27, a novel derivative of 4-hydroxyquinoline-3-formamide, prevents the development of murine systemic lupus erythematosus-like diseases in MRL/lpr autoimmune mice and BDF1 hybrid mice

**DOI:** 10.1186/ar4078

**Published:** 2012-11-01

**Authors:** Zhi-Yong Xiao, Shao-Hui Chen, Jun-Ping Cheng, Wen-Xia Zhou, Yong-Xiang Zhang, Ri-Fang Yang, Liu-Hong Yun

**Affiliations:** 1 Beijing Institute of Pharmacology and Toxicology, 27 Taiping Road, Haidian District, Beijing 100850, People's Republic of China

## Abstract

**Introduction:**

Naturally occurring CD4^+^CD25^+ ^regulatory T (Treg) cells are central to the maintenance of peripheral tolerance. Impaired activity and/or a lower frequency of these cells lead to systemic lupus erythematosus (SLE). Manipulating the number or activity of Treg cells is to be a promising strategy in treating it and other autoimmune diseases. We have examined the effects of Y27, a novel derivative of 4-hydroxyquinoline-3-formamide, on SLE-like symptoms in MRL/*lpr *autoimmune mice and BDF1 hybrid mice. Whether the beneficial effect of Y27 involves modulation of CD4^+^CD25^+ ^Treg cells has also been investigated.

**Methods:**

Female MRL/*lpr *mice that spontaneously develop lupus were treated orally by gavage with Y27 for 10 weeks, starting at 10 weeks of age. BDF1 mice developed a chronic graft-versus-host disease (GVHD) by two weekly intravenous injections of parental female DBA/2 splenic lymphocytes, characterized by immunocomplex-mediated glomerulonephritis resembling SLE. Y27 was administered to chronic GVHD mice for 12 weeks. Nephritic symptoms were monitored and the percentage of CD4^+^CD25^+^FoxP3^+ ^Treg peripheral blood leukocyte was detected with mouse regulatory T cell staining kit by flowcytometry. Purified CD4^+^CD25^+ ^Tregs were assessed for immune suppressive activity using the mixed lymphocyte reaction.

**Results:**

The life-span of MRL/lpr mice treated with Y27 for 10 weeks was significantly prolonged, proteinuria and renal lesion severity were ameliorated, and blood urea nitrogen, triglyceride and serum anti-double-stranded DNA antibodies were decreased. Similar results were found in chronic GVHD mice. Administration of Y27 had little impact on percentage of the peripheral blood lymphocyte CD4^+^CD25^+^Foxp3^+ ^Treg cells in both groups of mice. In contrast, the suppressive capacity of CD4^+^CD25^+ ^Treg cells in splenocytes was markedly augmented in Y27-treated mice *ex vivo*.

**Conclusions:**

Experimental evidence of the protect effects of Y27 against autoimmune nephritis has been shown. The mechanism may involve enhancement of the suppressive capacity of CD4^+^CD25^+ ^Treg cells.

## Introduction

Systemic lupus erythematosus (SLE) is a disorder of immune regulation characterized by the breakdown of tolerance to self-nuclear, cytoplasmic and cell surface molecules, and the production of autoantibodies to them. Antibody- and immune complex-mediated inflammation in SLE can lead to the development of glomerulonephritis, dermatitis, serositis, and vasculitis [1]. The autoimmune MRL/*lpr *mouse substrain spontaneously develops a severe disease with many symptoms closely resembling human SLE, that is, hypergammaglobulinemia, various autoantibodies, and glomerulonephritis [2]. Murine chronic graft-versus-host disease (GVHD) is a well-established lupus model induced by transferring DBA/2 parental spleen cells into (C57BL/6 × DBA/2) F1 (BDF1) mice. BDF1 mice develop a systemic autoimmune disorder resembling human SLE, characterized by autoantibody production, immunocomplex deposition and proteinuria [3-5]. In both these models, an abnormal function of CD4^+^CD25^+ ^regulatory T (Treg) cells may play a pivotal role.

Naturally arising CD4^+ ^Treg cells expressing the IL-2 receptor α-chain (CD25) and the transcription factor forkhead box P3 (FoxP3) represent a subset of thymus-derived CD4^+ ^T cells critical for the control of most immune responses, including autoimmunity, transplantation tolerance, antitumor immunity and anti-infectious reactions [6,7]. Treg cells fail to proliferate or secrete cytokines in response to polyclonal or antigen-specific stimulation, but can inhibit the proliferation and activation of conventional CD4^+^CD25^- ^effector T cells (Tconv) as well as CD8^+ ^T cells [8]. The mechanisms by which Treg cells mediate their suppressive effects have not been fully elucidated. Treg cells suppress immune responses through contact-dependent mechanisms and the production of soluble factors, including transforming growth factor β (TGF-β) and IL-10 [9-13]. Quantitative and/or qualitative deficiencies of Treg cells are considered responsible for a situation where the sum of autoreactive effector T-cell responses overwhelms the capacity of a weakened Treg compartment, thereby triggering overt autoimmune disease [14]. Although there are some discrepant reports (possibly due to variations in CD4^+^CD25^+ ^T cell analysis), studies in patients with SLE show that CD4^+^CD25^+ ^Treg cell numbers are reduced and suppressive functions are compromised when tested *ex vivo *[15]. Similar defects have been found in lupus models. In lupus-prone MRL/*lpr *mice developing a strong lupus disease, a reduced capacity to suppress proliferation and especially, to inhibit interferon-γ (IFN-γ) secretion by syngeneic effector CD4^+^CD25^- ^T cells occurs *in vitro *[16]. In BDF1 mice, infusion of purified Treg cells at the time of transplant can prevent the development of lethal GVHD, whereas depletion makes matters worse [17-19]. Therefore, expanding Treg or enhancing Treg suppressive activity *in vivo *offers a promising strategy in lupus treatment.

**Figure 1:**
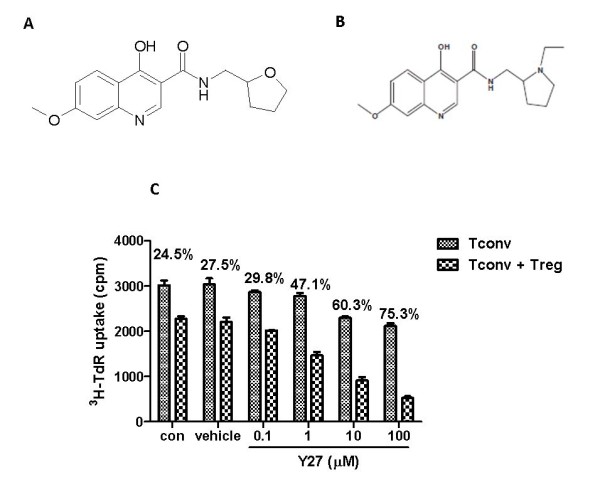
**Structure of Y27 and its effect on CD4^+^CD25^+ ^regulatory T (Treg) cells**. (**A**) Structure of Y27. (**B**) Structure of H1521. (**C**) Y27 concentration-dependent enhancement of the suppressive capacity of CD4^+^CD25^+ ^Treg cells in C57BL/6 mice *in vitro*. Suppressive activity of CD4^+^CD25^+ ^Treg cells was expressed as percent suppression calculated as following: 100 × (cpm (CD4^+^CD25^- ^Tconv) - cpm (CD4^+^CD25^- ^Tconv + CD4^+^CD25^+ ^Treg))/cpm (CD4^+^CD25^- ^Tconv). Tconv, conventional CD4^+^CD25^- ^effector T cells.

Y27 is a novel 4-hydroxyquinoline-3-formamide derivative primarily derived from H1521, which could ameliorate glomerular injury in the chronic GVHD murine model [20] (Y27, Figure 1A; H1521, Figure 1B). Y27 and H1521 are both 4-hydroxy-7-methoxyquinoline-3-carboxamide. Y27 differs from H1521 in that the N-substituent is tetrahydrofuran-2-methyl instead of 1-ethyl-tetrahydropyrrol-2-methyl of H1521, that is, non-basic oxygen bioisosterically replaces the basic nitrogen. Thus, Y27 merely has one chiral center of 2-carbon with two enantiomers and this is absent in the second chiral center of tertiary nitrogen of H1521. This makes the Y27 product simple and in control (H1521's chemico-physical properties, including solubility and pharmacokinetic characters, might change under different storage conditions and time).

Preliminary studies showed that Y27 could boost the suppressive capacity of CD4^+^CD25^+ ^Treg cells in C57BL/6 mice assessed *in vitro *by the mixed lymphocyte reaction (MLR) (Figure 1C). We have focused on the protection effect of Y27 against autoimmune nephritis in MRL/*lpr *mice and BDF1 cGVHD mice. The influence of Y27 on CD4^+^CD25^+ ^Treg cells *ex vivo *was also followed. Y27 treatment effectively ameliorated autoimmune syndromes in MRL/*lpr *mice and BDF1 mice, which might be the consequence of an enhanced suppressive capacity of CD4^+^CD25^+ ^Treg cells.

## Materials and methods

### Animals

Female MRL/Mp, MRL/*lpr*, CBA/Ca mice (6 to 8 weeks old) were purchased from Shanghai SLAC Laboratory Animal Co. Ltd (Shanghai, China). Female DBA/2, BDF1 and C57BL/6 mice (8 to 10 weeks old) were purchased from Vitalriver Experimental Animal Center (Beijing, China). Experiments were carried out in accordance with the National Institutes of Health Guidelines for Care and Use of Laboratory Animals, and were approved by the Bioethics Committee of the Beijing Institute of Pharmacology and Toxicology. All husbandry and experimental contact with the mice was carried out under specific pathogen-free conditions. The mice were allowed to acclimatize in the facility for 1 week before experiments began.

### Reagents

Horseradish peroxidase (HRP)-goat anti-mouse IgG was purchased from Rockland Immunochemicals Inc, Gilbertsville, PA, USA. Anti-mouse IgG goat polyclonal antibody was purchased from Santa Cruz Biotechnology Inc, Santa Cruz, CA, USA. Calf thymus DNA and 3, 3', 5, 5'-tetramethylbenzidine (TMB) were from Sigma-Aldrich, St. Louis, MO, USA. RPMI 1640 medium and fetal bovine serum (FBS) were purchased from GibcoBRL Life Technologies, Grand Island, NY, USA. Mouse CD4^+^CD25^+ ^Treg cell isolation kit was obtained from Miltenyi Biotec GmbH, Bergisch Gladbach, Germany. The mouse Treg cell staining kit came from eBioscience, San Diego, CA USA, and contained anti-mCD16/CD32, fluorescein isothiocyanate (FITC)-conjugated anti-mCD4 (RM4-5), allophycocyanin (APC)-conjugated anti-mCD25 (PC61.5), phycoerythrin (PE)-conjugated FoxP3 (FJK-16s), with the required buffers. Mouse TGF-β1, IL-2 and IFN-γ ELISA kits were purchased from Dakewe Biotech co., Ltd, Shenzhen, China.

### MRL/*lpr *mice experiment protocol

Six-week-old MRL/*lpr *mice were monitored for the development of nephritis by measuring urine protein weekly. Until 10 weeks of age, approximately 50% of the urine protein samples exceeded 2 mg/ml. To investigate the protective effects of Y27, MRL/*lpr *mice were randomly divided into five groups (*n *= 6 or 10 mice per group) according to body weight and urine protein level as follows: vehicle control (saline), cyclophosphamide (CYC) 15 mg/kg/day, or Y27 10, 20, 40 mg/kg/day by gavage. The experiment was terminated after 10 weeks when all MRL/*lpr *mice treated with vehicle control had developed 1^+ ^proteinuria (urine protein > 3 mg/ml). For mice that died before termination, the last known values for urinary protein were carried forward. The experiments were repeated twice.

### Induction of chronic GVHD and Y27 treatment

Chronic GVHD was induced in female BDF1 mice by two weekly intravenous (iv) injections of 5 × 10^7 ^parental female DBA/2 splenic lymphocytes. The BDF1 mice were divided into five groups (*n *= 7 mice per group) according to body weight and urine protein level, as follows: vehicle control (saline) and Y27, 10, 20, 40 mg/kg/day. Age- and sex-matched F1 mice serving as controls were injected iv with the same volume of Hank's buffer. Y27 was dissolved in saline and given by gavage once a day from 3 days after the last cell injection, continuing for 12 weeks. Normal control and GVHD control mice received the same dose of vehicle.

### Proteinuria, blood urea nitrogen and triglyceride

Blood and urine samples were collected every other week for assay. Mice were placed in metabolic cages for a 24-h collection of urine, and urinary protein excretion was determined using the Bradford assay. Urinary protein was scored according to the following criteria: 0 < 3 mg/ml; 1^+^, 3 mg/ml; 2^+^, 10 mg/ml; 3^+^, 30 mg/ml; and 4^+^, 100 mg/ml. Serum blood urea nitrogen (BUN) and triglyceride levels were measured with an automatic biochemical analyzer (HITACHI, 7020).

### Measurement of serum autoantibodies and immunogIoblin

Anti-double-stranded DNA (anti-dsDNA) antibody and immunoglobulin G (IgG) levels in serum were measured using immunoassay as follows: microtiter plates were coated with calf thymus DNA or goat anti-mouse IgG, respectively. HRP-conjugated goat anti-mouse IgG (Santa Cruz, CA, USA), 3,3'-5,5' tetramethylbenzidine (TMB) and H_2_O_2 _were added in that order. The reaction was stopped with 2M H_2_SO_4 _and the plates were read at 450 nm in a Titertek Multiskan photometer (Netherlands).

Serum IgG1 and IgG2a levels were determined by sandwich ELISA. Briefly, microtiter plates were coated with rabbit polyclonal antibody against mouse IgG (Santa Cruz, CA, USA). Goat anti-mouse IgG1 or IgG2a were used for primary Abs, with HRP-conjugated rat anti-goat IgG (Santa Cruz, CA, USA) as the secondary Ab for ELISA.

### Renal histopathology

Mice were sacrificed at the end of the study for histological evaluation of the kidneys. The left kidney from each mouse was removed, fixed in 4% formalin, and embedded in paraffin. Five-micrometer sections were stained with hematoxylin-eosin. An observer who was blind to the source of each section scored glomeruli and classified the lesion [21]. The following scale was applied: 0, normal morphology; 1, moderate expansion of the glomerular matrix without glomerulonephritis; 2, mild glomerulonephritis with mesangial hypercellularity and/or segmental necrosis; 3, severe glomerulonephritis with extensive sclerosis and/or loop necrosis and/or cellular crescent. A score was assigned to each of >100 glomeruli/ mouse, and the mean calculated for each group.

### Flow cytometry

A CD4^+^CD25^+^FoxP3^+ ^population assay was carried out with mouse Treg cell staining kit. Briefly, murine peripheral blood lymphocytes were collected and blocked with affinity purified anti-mCD16/CD32 (Fc block), stained with FITC-conjugated anti-mCD4 (RM4-5), APC-conjugated anti-mCD25 (PC61.5), and PE-conjugated FoxP3 (FJK-16s). Samples were analyzed in a FACSCalibur system with CellQuest software (BD, San Jose, CA, USA).

### CD4^+^CD25^- ^and CD4^+^CD25^+ ^T cells preparation

Mice were sacrificed and the spleens removed aseptically. A single splenocyte suspension was prepared, from which cell debris and clumps were removed. Erythrocytes were lysed with Tris-buffered ammonium chloride (0.155 M NH_4_Cl and 16.5 mM Tris, pH 7.2). Mononuclear cells were washed and resuspended in RPMI 1640 medium containing 10% FBS. CD4^+^CD25^- ^and CD4^+^CD25^+ ^T cells were isolated with mouse CD4^+^CD25^+ ^Treg cell isolation kit. Briefly, CD4^+ ^T cells were firstly pre-enriched by depleting unwanted cells using negative selection, and CD25^+ ^cells were isolated from the CD4^+ ^cell population by staining with PE-labeled anti-CD25 mAb followed by incubation with magnetic-activated cell sorting (MACS) anti-PE microbeads. CD4^+^CD25^+ ^T cells were positively selected on a MACS mini-separation magnetic column, from which the flow-through fraction containing CD4^+^CD25^- ^T cells was collected. The purity of cell subsets was > 90%, as determined by fluorescence activated cell sorter (FACS) analysis.

### Suppression assay

The suppressive capacity of CD4^+^CD25^+ ^Treg cells was assessed by the mixed lymphocyte reaction. CD4^+^CD25^- ^T cells were cultured (2 × 10^4 ^cells/well) in plates pre-coated with anti-CD3 mAb (1 μg/ml) in the presence of antigen-presenting cells (5 × 10^4^/well T cell-depleted and mitomycin C-treated splenic cells) [16], with or without CD4^+^CD25^+ ^T cells (10^4^/well). Cultures were prepared in complete RPMI 1640 medium supplemented with 10% FBS. To measure proliferation, ^3^H-thymidine (0.5 μCi, specific activity 6.7 Ci/mmole) was added after 56 h and the cells harvested 16 h later on a glass filter. The radioactivity incorporated was determined with a Beta Scintillation Counter (MicroBeta Trilux, Wellesley, MA, USA). Suppressive activity of CD4^+^CD25^+ ^Treg cells was expressed as percent suppression calculated as following:

$$\text{1}00\times \left(\text{cpm }\left(\text{CD}{\text{4}}^{+}\text{CD2}{\text{5}}^{-}\text{Tconv}\right)\;-\text{ cpm }\left(\text{CD}{\text{4}}^{+}\text{CD2}{\text{5}}^{-}\text{Tconv }+\text{ CD}{\text{4}}^{+}\text{CD2}{\text{5}}^{+}\text{Treg}\right)\right)/)\text{cpm }\left(\text{CD}{\text{4}}^{+}\text{CD2}{\text{5}}^{-}\text{Tconv}\right).$$

### Cytokine analysis

The levels of TGF-β1 in the culture supernatants were determined by ELISA, and the results were expressed as the cytokine concentration pg/ml, with the detection limit set at15 pg/ml.

### Statistical analysis

Unless otherwise stated, data are expressed as mean ± standard error of the mean (SEM). One-way analysis of variance (ANOVA) was used to assess significant differences between groups. Dunnett's multiple comparisons test was used to test significant effects among multiple group means.

## Results

### Effect of Y27 on proteinuria, anti-dsDNA antibody, IgG levels and kidney histological score in MRL/*lpr *mice

**Figure 2:**
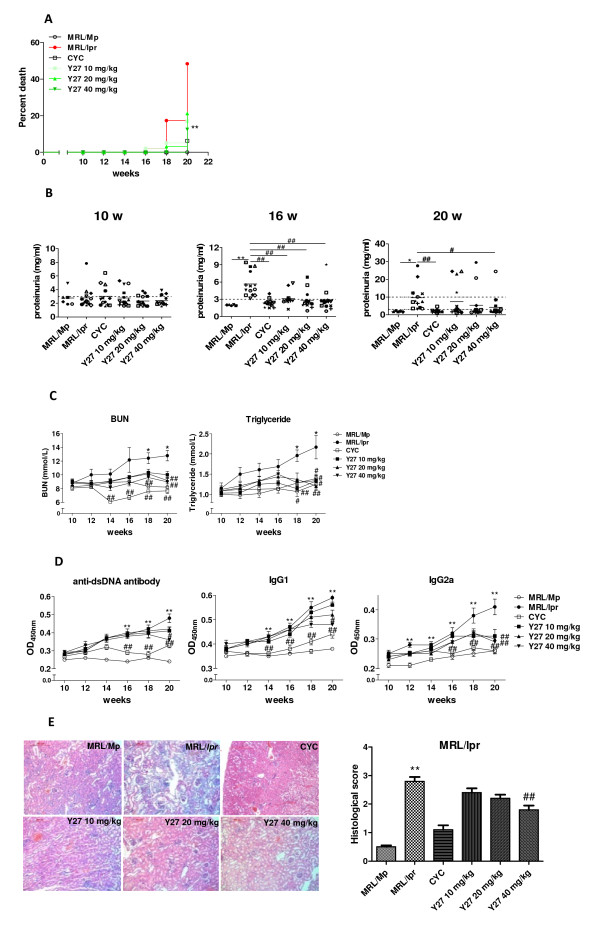
**Y27 treatment of lupus syndromes, and effects on serum antibody and renal histology in MRL/*lpr *mice**. MRL/*lpr *mice (10 weeks old) were treated with vehicle control (saline), cyclophosphamide (CYC; 15 mg/kg), or Y27 (10, 20 and 40 mg/kg) (*n *= 16 per group). The experiment was terminated after 10 weeks of treatment when all MRL/*lpr *mice treated with vehicle control developed 1^+ ^proteinuria (urine protein > 3 mg/ml). (**A**) Effect of treatment on mortality over 10 weeks (analyzed by log rank test) and urinary protein levels at 10, 16 and 20 weeks of age. Each symbol represents one mouse. Bars show the mean of the group. (**B**) Effect of treatment on blood urea nitrogen and triglyceride levels. (**C**) Anti-double-stranded DNA (anti-dsDNA) antibodies, IgG1and IgG2a levels after Y27 treatment. (**D**) Y27 attenuation of renal histopathological changes in MRL/*lpr *mice (HE stain, 100×). For the blinded scoring of glomerular alterations see Materials and methods).*P < 0.05; **P < 0.01 versus MRL/Mp. ^#^*P *< 0.05; ^##^*P *< 0.01 versus MRL/*lpr*.

Generally, 50% mortality occurred at the 5^th ^month of the life-span in MRL/*lpr *mice [22]. Figure 2A shows that 48.3% MRL/*lpr *mice died at the 20^th ^week (10 weeks after Y27 administration), while Y27 significantly increased mice survival. These experiments were repeated twice, giving identical results, and thus the data has been pooled (*n *= 16/group). At 10 weeks of age, only half of MRL/*lpr *mice had low proteinuria levels (Figure 2A). With aging, proteinuria in vehicle-treated mice progressively increased, but the onset of severe proteinuria was significantly delayed in both Y27- and CYC-treated mice. At 16 weeks of age, all mice in the vehicle-treated group developed 1^+ ^proteinuria compared with two of sixteen mice in the high dose Y27-treated group. At 20 weeks of age, five mice developed 2^+ ^proteinuria, and other mice developed 1^+ ^proteinuria in the vehicle-treated group compared with one mouse that developed 2^+ ^proteinuria, and with two mice that developed 1^+ ^proteinuria after high dose Y27 treatment. Except for the three mice that developed proteinuria as mentioned, two mice died during the experiment, while the remaining eleven in this group did not develop proteinuria (Figure 2A). At the end of treatment, mice also had significantly elevated serum BUN and triglyceride from 18 weeks of age, while treatment with 40 mg/kg Y27 significantly improved the situation (Figure 2B).

The presence of high avidity IgG autoantibodies to dsDNA tends to be correlated with SLE disease activity. The levels of serum anti-dsDNA antibody in our investigation significantly increased from 16 weeks of age in MRL/*lpr *mice, remaining high until the end of the experiment. Y27, given at 40 mg/kg, decreased serum anti-dsDNA antibody level (Figure 2C). Moreover, as an important immunological parameter, serum IgG1 and IgG2a levels were markedly elevated in MRL/*lpr *mice. Y27 significantly decreased IgG1 and IgG2a levels at both 20 and 40 mg/kg (Figure 2C).

**Figure 3:**
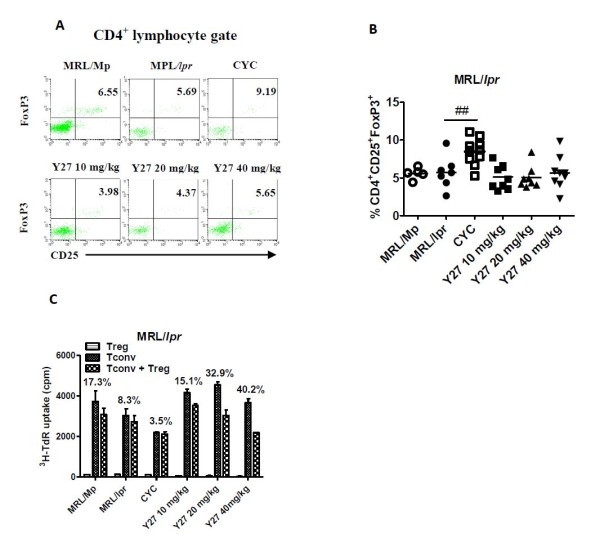
**Y27 treatment influence on the percentage of CD4^+^CD25^+^FoxP3^+ ^regulatory T (Treg) cells in peripheral blood, and the suppressive capacity of CD4^+^CD25^+ ^Treg cells in MRL/*lpr *mice**. MRL/*lpr *mice (10 weeks old) were treated with vehicle control (saline), cyclophosphamide (CYC; 15 mg/kg), or Y27 (10, 20 and 40 mg/kg) for 10 weeks (*n *= 7 to 10 per group). (**A**) and (**B**) The percentage of peripheral blood CD4^+^CD25^+^FoxP3^+ ^Treg cells is unaltered in Y27-treated MRL/*lpr *mice. (**C**) Suppressive capacity of CD4^+^CD25^+ ^Treg cells is significantly increased after Y27 treatment Percent suppression = 100 × (cpm (CD4^+^CD25^- ^Tconv) - cpm (CD4^+^CD25^- ^Tconv + CD4^+^CD25^+ ^Treg))/cpm (CD4^+^CD25^- ^Tconv). Results represent mean ± standard error of the mean (SEM). ^##^*P *< 0.01 versus MRL/*lpr*. Tconv, conventional CD4^+^CD25^- ^effector T cells.

Proteinuria is the direct result of renal damage, the severity being assessed histologically by a board-certified pathologist using the scoring method outlined in the Methods and Material section, as described by Senuma *et al. *[21]. The mice remaining at the end of the experiment were analyzed for the presence and extent of glomerulonephritis by conventional histology. Figure 3B shows the histological score of MRL/*lpr *mice as > 3, implying that extensive sclerosis and/or loop necrosis and/or cellular crescent appeared in almost every glomerulus. Y27 treatment, notably a high dose regimen, reduced the severity of the renal histopathology as compared to vehicle-treated mice (approximately 1.5- to 1.3-fold decrease in average score compared to vehicle, Figure 3E).

### Effect of Y27 on the quantity and suppressive capacity of CD4^+^CD25^+^FoxP3^+ ^Treg cells in MRL/*lpr *mice

We had noted from earlier studies that Y27 could enhance the suppressive capacity of CD4^+^CD25^+ ^Treg cells in C57BL/6 mice assessed by MLR without augmenting the CD4^+^CD25^+^FoxP3^+ ^population (Figure 1C). Here, we first examined the percentage of Treg cells in MRL/*lpr *mice by flow cytometry. As before, MRL/*lpr *mice had a normal percentage of CD4^+^CD25^+^FoxP3^+ ^T cells in the peripheral blood (5.59% versus 5.73%, Figure 3A, 3B). CYC treatment significantly augmented the Treg population (8.49%, *P *< 0.01), but simultaneously reduced peripheral blood leukocyte count (data not shown). Thus, the number of CD4^+^CD25^+^FoxP3^+ ^T cells remained unaltered in CYC-treated mice. Unlike CYC, Y27 treatment affected neither the percentage of Treg cells (5.14%, 5.07%, 5.66%, Figure 3A, 3B) nor peripheral blood leukocyte count (data not shown). Likewise, the quantity of CD4^+^CD25^+^FoxP3^+ ^T cells was unchanged in either of the Y27-treated mice.

Regarding quantitative analysis of Treg cells, we have investigated the regulatory properties of CD4^+^CD25^+ ^Treg by incubating CD4^+^CD25^- ^effector T cells, stimulated with both anti-CD3 mAb and haplotype-matched antigen presenting cells, with CD4^+^CD25^+ ^T cells (ratio 2:1). CD4^+^CD25^+ ^T cells purified from MRL/*lpr *mice displayed a non-proliferation phenotype when stimulated by anti-CD3 mAb and autologous antigen presenting cells (Figure 3C). Under these activation conditions, a reduced capacity of MRL/*lpr *Treg cells to inhibit the proliferation of syngeneic effector T cells was seen compared with co-cultures with MRL/Mp Treg cells and effector T cells (8.3% versus 17.3%, *P *< 0.05). CYC treatment seemed to reduce further the suppressive capacity of Treg cells, which affected CD4^+^CD25^- ^effector T cell proliferation more markedly than CD4^+^CD25^+ ^T cells. However, an apparent recuperative capacity of Treg cells to inhibit the proliferation of effector T cells was seen in Y27-treated mice. Notably with 20 and 40 mg/kg Y27, the suppressive capacity of MRL/*lpr *Treg cells was restored to syngeneic co-cultures with CBA/Ca Treg cells and effector T cells (approximately 35%).

### Effect of Y27 on cytokine TGF-β1 and IL-10 production *ex vivo *in MRL/*lpr *mice

**Figure 4:**
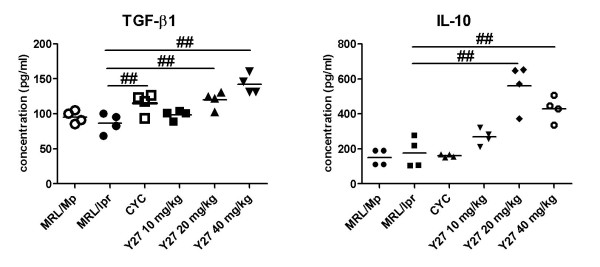
**Y27 treatment promotion of transforming growth factor (TGF)-β1 and interleukin (IL)-10 secretion by purified CD4^+^CD25^+ ^T regulatory (Treg) cells in MRL/*lpr *mice**. MRL/*lpr *mice (10 weeks old) were treated with vehicle control (saline), cyclophosphamide (CYC; 15 mg/kg), or Y27 (10, 20 and 40 mg/kg) for 10 weeks. (*n *= 4). Results represent mean ± standard error of the mean (SEM). ^##^*P *< 0.01 versus MRL/*lpr*.

To examine the possible path by which Y27 enhances the suppressive capacity of CD4^+^CD25^+ ^Treg cells, the key cytokines TGF-β1 and IL-10 *ex vivo *were detected in MRL/*lpr *mice, since they play an important role in Treg inhibition. TGF-β1 and IL-10 levels in the supernatants of CD4^+^CD25^+ ^T cells stimulated with anti-CD3 mAb were only mildly decreased in MRL/*lpr *mice compared with MRL/Mp mice, whereas CYC and Y27 significantly increased TGF-β1 and IL-10 production (Figure 4).

### Effect of Y27 on proteinuria, anti-dsDNA antibody, IgG levels and kidney histological score in BDF1 mice

**Figure 5:**
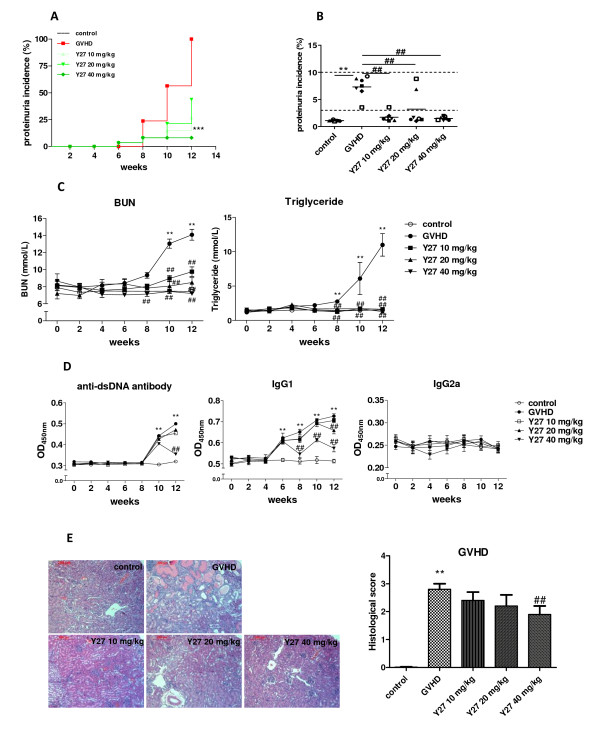
**Y27 treatment amelioration of lupus syndromes, and effects on serum antibody and renal histology in BDF1 mice**. Chronic graft-versus-host disease (GVHD) was induced in recipient BDF1 mice by two intravenous injections of donor parental DBA/2 mice lymphocytes. Y27 (10, 20 and 40 mg/kg) was given from 3 days after the last cell injection, the control and model mice being given the same dose of vehicle (*n *= 7 per group). (**A**) Effect of treatment on mortality over 10 weeks (analyzed by log rank test) and urinary protein levels at the end of the experiment. Each symbol represents one mouse. Bars show the mean of the group. (**B**) Effect of treatment on blood urea nitrogen and triglyceride levels. (**C**) Anti-dsDNA antibodies and IgG1 levels decreased after Y27 treatment. (**D**) Y27 attenuated renal histopathology change in BDF1 mice (HE stain, 100×). Blinded scoring of glomerular alterations was done as indicated in Materials and methods. Bars show the mean ± standard error of the mean (SEM). **P < 0.01 versus control; ^#^*P *< 0.05, ^##^*P *< 0.01 versus GVHD.

In BDF1 mice, 1^+ ^proteinuria occurred from the 8th week after BDF1 recipients were injected with DBA/2 lymphocytes (Figure 5A). At the 12th week, all the mice treated with vehicle developed proteinuria, whereas onset was significantly postponed in Y27-treated mice (Figure 5A). In the Y27 40 mg/kg group, none of the mice developed proteinuria. BUN and triglyceride levels were also significantly increased in GVHD mice at 8 to 12 weeks after cell transfer, whereas Y27 treatment lowered them (Figure 5B).

In BDF1 mice, serum anti-dsDNA antibody was markedly elevated from 10 weeks after the second cell injection, which was sustained until the 12th week. Y27 inhibited the rise of serum anti-dsDNA antibody, the effect being statistically significant at 40 mg/kg compared with the control group (Figure 5C). Among serum IgG subtypes, IgG1 was preferentially elevated from 6 weeks after sensitization, whereas IgG2a was barely changed in chronic GVHD controls. Y27 significantly decreased IgG1 level at 40 mg/kg, with little influence on IgG2a level (Figure 5C).

Chronic GVHD resulted in the development of immunocomplex-mediated glomerulonephritis, the pathological changes being similar to those in lupus nephritis; the changes were noted about the 12th week after GVHD induction. The renal histopathology score was significantly higher in the GVHD model group (2.8 ± 0.2) than in the control group. Y27 treatment at 10, 20, and 40 mg/kg lowered the scores to 2.4 ± 0.3, 2.2 ± 0.4 and 1.9 ± 0.3, respectively (Figure 5D).

### Effect of Y27 on the quantity and suppressive capacity of CD4^+^CD25^+^FoxP3^+ ^Treg cells in BDF1 mice

**Figure 6:**
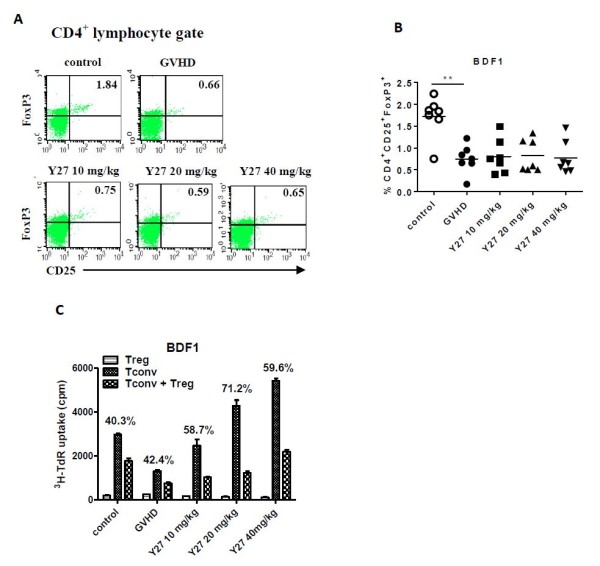
**Y27 treatment effect on the percentage of CD4^+^CD25^+^FoxP3^+ ^regulatory T (Treg) cells in peripheral blood, and the suppressive capacity of CD4^+^CD25^+ ^Treg cells in BDF1 mice**. Chronic graft-versus-host disease (GVHD) was induced in recipient BDF1 mice by two intravenous injections of donor parental DBA/2 mice lymphocytes. Y27 (10, 20 and 40 mg/kg) was given from 3 days after the last cell injection. The control and model mice received the same dose of vehicle (*n *= 7 per group). (**A**) and (**B**) Y27 treatment affect the percentage of peripheral blood CD4^+^CD25^+^FoxP3^+ ^Treg cells. (**C**) Effect of Y27 treatment on the suppressive capacity of CD4^+^CD25^+ ^Treg cells. Percent suppression = 100 × (cpm (CD4^+^CD25^- ^Tconv) - cpm (CD4^+^CD25^- ^Tconv + CD4^+^CD25^+ ^Treg))/cpm (CD4^+^CD25^- ^Tconv). Results represent mean ± standard error of the mean (SEM). **P < 0.01 versus control. Tconv, conventional CD4^+^CD25^- ^effector T cells.

In BDF1 mice, CD4^+^CD25^+^FoxP3^+ ^Treg cells represented 1.72% of peripheral blood leukocytes in the control group, whereas in sensitized BDF1 mice there was a significant decrease (0.75%) (Figure 6A, B). This decrease was also partly compromised by increase in the peripheral blood leukocyte count (data not shown). Y27 treatment did not influence the CD4^+^CD25^+^FoxP3^+ ^Treg population at any of the three doses (0.80%, 0.82%, 0.77% respectively, Figure 6A, B), nor did it influence the leukocyte count in peripheral blood compared with cGVHD mice (data not shown).

An analogous assessment of the suppressive properties of CD4^+^CD25^+ ^Treg cells was also conducted in BDF1 mice. Upon activation with anti-CD3 mAb and autologous antigen presenting cells, there was no clear difference in the suppressive properties between the control and the GVHD groups (40.3% versus 42.4%, Figure 6C). However, CD4^+^CD25^+ ^Treg cells from Y27-treated GVHD mice had enhanced suppressive activity, particularly at 20 and 40 mg/kg (71.24%, *P *< 0.01; 59.58%, *P *< 0.05).

## Discussion

The novel compound Y27 showed potent immunosuppressive activity both *in vitro *and *in vivo*, including enhanced suppressive capacity of purified CD4^+^CD25^+ ^Treg cells in preliminary screenings. In the present study, our results indicate that Y27 treatment strongly prevented the development of proteinuria and nephritis symptoms, decreased serum autoantibody production, ameliorated lethal renal injury, and consequently prolonged the lifespan of both lupus-prone types of mice. The therapeutic effects of Y27 may, at least partially, contribute to the restoration of the suppressive activity of Treg cells.

Autoimmunity can result from a loss of regulation of autoreactive T cells. CD4^+^CD25^+ ^Treg cells are of paramount importance in the maintenance of peripheral self-tolerance and avoidance of autoimmunity [23,24]. However, defects in the number and function of Treg cells, as well as a resistance of effector T cells to Treg cell-mediated suppression, could each contribute to failure in T cell regulation [15,16]. Each of these defects appears to contribute to the development of autoimmunity in several models. The underlying mechanisms by which these defects in regulation occur in lupus models have also been investigated. A deficiency of Treg cell numbers in two murine models, (NZB × NZW) F1 and (SWR × NZB) F1 has been noted [22]. However, MRL/*lpr *lupus mice, in which a fatal immune complex glomerulonephritis develops, are not deficient in CD4^+^CD25^+^FoxP3^+ ^Treg cells compared with non-autoimmune mice [16]. Consistent with this report, MRL/*lpr *mice had a normal percentage of CD4^+^CD25^+^FoxP3^+ ^T cells in the peripheral blood in our study. The chemotherapeutic alkylating agent, CYC, is widely used to treat autoimmune disorders with a dose-dependent bimodal effect on the immune system. CYC reduces the number of Treg cells in healthy mice; the changes found by others were minor and short-lived [25,26]. Our data indicate that the absolute number of CD4^+^CD25^+^FoxP3^+ ^T cells was unaltered after CYC treatment, although the percentage Treg cells in the CD4^+ ^population increased. This might be due to leucopenia caused by CYC. Y27 did not affect either the percentage of CD4^+^CD25^+^FoxP3^+ ^T cells or the peripheral blood leukocyte count.

In addition to a numerical deficiency of CD4^+^CD25^+ ^Treg cells contributing to the pathogenesis of SLE, functional abnormalities of Treg cells may also exist. While MRL/Mp CD4^+^CD25^+ ^Treg cells show only subtle abnormalities of regulatory function, MRL/*lpr *CD4^+^CD25^+ ^Treg cells have a distinctly reduced capacity to inhibit the proliferation of effector T cells [16]. In concordance with these results, we have demonstrated that a more severe decrease in Treg cell function can be seen in MRL/*lpr *mice compared to MRL/Mp mice. CYC treatment leads to a further decrease in Treg cell function [25,27]. However, Y27 increases the suppressive capability of Treg cells quite remarkably. It remains difficult to determine whether the increase in Treg cell inhibition in MRL/*lpr *mice is due to an enhanced competence in the CD4^+^CD25^+ ^T cell population or sensitization of responder CD4^+^CD25^- ^T cells themselves to be suppressed. To clarify this issue, a series of crossover experiments will be necessary.

Inadequate soluble cytokines TGF-β and IL-10 mostly contribute to defective Treg cell function [9-11]. The suppressive effects of TGF-β can be transmitted to effector T cells through its soluble forms, or its direct contact with Tregs, which display TGF-β on their surface [28]. When cell-to-cell contact takes place, TGF-β molecules on the surface of Tregs aggregate, and this is triggered by signals emanating from cytolytic T-lymphocyte-associated antigen-4 (CTLA-4) [28]. IL-10 is another important Treg-associated cytokine that might regulate the pathogenesis of SLE. It impedes the activation/expansion of autoreactive lymphocytes by preventing the activation of antigen presenting cells and downregulating the expression of co-stimulatory molecules [29]. Furthermore, IL-10 may play a role in Treg commitment and function [29]. In our study, an increase of both TGF-β and IL-10 was found in the supernatants of Treg cells after Y27 treatment. We speculate that elevated TGF-β1 and IL-10 production by Treg cells contributes, at least partially, to enhanced suppressive capacity of Treg cells facilitated by Y27.

In another lupus-prone BDF1 mouse, immune tolerance to self-antigens may be defective, while onset of chronic GVHD gives rise to autoimmune manifestations in the disorder. Recent attention has focused on CD4^+^CD25^+ ^Treg cells and their relationship to chronic GVHD [30]. Treg number measured by CD4^+^CD25^+^FoxP3^+ ^staining has been found to be decreased in patients with chronic GVHD [31-33]. Moreover, Treg number was found to return to normal in patients with resolved chronic GVHD. Our chronic GVHD mice showed a robust decrease in the percentage of CD4^+^CD25^+^FoxP3^+ ^T cells, which was compromised by an expanded T cell population. When purified, these Treg cells had considerable suppressive ability *in vitro*. Unlike current immunosuppressants, such as sirolimus, which exert their effect through expansion of Treg [34], Y27 had little effect on Treg number. However, Y27 greatly increased the suppressive capability of Treg cells, and the mechanism(s) involved are now under investigation.

A novel oral quinoline-3-carboxamide derivative, laquinimod, is being assessed in the treatment of relapsing-remitting (RR) multiple sclerosis (MS) and other autoimmune diseases [35-37]. Laquinimod is structurally related to roquinimex (linomide), which has been demonstrated to have efficacy in MS [38], although its development was halted after unexpected serious adverse events in a phase III trial [39]. However, Laquinimod has since shown efficacy in phase II and phase III MS clinical trials without evident immunosuppression or significant toxicity [35,36,40]. The precise mechanisms of action of laquinimod have yet to be elucidated; however, current studies in the MS model, experimental autoimmune encephalomyelitis (EAE), indicate that laquinimod can suppress immune cell migration and expression of inflammatory cytokines, including IFN-γ, TNF-α, IL-13, and IL-17 [41]. In addition, laquinimod can increase CD4^+^CD25^+^Foxp3-expressing Treg cells in spleens of C57BL/6 mice immunized with MOG p35-55. Moreover, Foxp3-expression was induced by laquinimod by direct action on antigen-presenting cells function. When purified laquinimod-treated splenic CD11b^+^CD11c^- ^cells were used as antigen-presenting cells in co-culture with naïve MOG p35-55-specific T cells, Foxp3-expressing CD4^+ ^T cells were significantly elevated compared with the vehicle control [42]. As studies of the use of laquinimod in lupus are few, we do not know whether it could affect Treg and antigen-presenting cells in lupus models. Sharing some structural similarity with laquinimod, Y27 might promote the suppression capacity of Y27 through altered antigen-presenting cell function, but this is a hypothesis that needs verification.

## Conclusions

In conclusion, our results provide direct evidence of the protective effects of the novel 4-hydroxyquinoline-3-formamide derivative, Y27, against two murine SLE-like disease models. In both spontaneous lupus-prone MRL/*lpr *mice and sensitized BDF1 mice, Y27 showed potent disease-modifying activity. The mechanism might involve enhancement of the suppressive capacity of CD4^+^CD25^+ ^Treg cells.

## Abbreviations

ANOVA: analysis of variance; anti-dsDNA: anti-double-stranded DNA; APC: allophycocyanin; BUN: blood urea nitrogen; CYC: cyclophosphamide; ELISA: enzyme-linked immunosorbent assay; FACS: fluorescence activated cell sorter; FBS: fetal bovine serum; FITC: fluorescein isothiocyanate; FoxP3: forkhead box P3; GVHD: graft-versus-host disease; HRP: horseradish peroxidase; IFN-γ: interferon-γ; IgG: immunoglobulin G; IL: interleukin; iv: intravenous; MACS: magnetic-activated cell sorting; MLR: mixed lymphocyte reaction; MS: multiple sclerosis; PE: phycoerythrin; SEM: standard error of the mean; SLE: systemic lupus erythematosus; Tconv: conventional CD4^+^CD25^- ^effector T cells; TGF-β: transforming growth factor β; TMB: 3, 3', 5, 5'-tetramethylbenzidine; TNF-α: tumor necrosis factor-α; Treg: regulatory T.

## Competing interests

Beijing Institute of Pharmacology & Toxicology holds the patent of the title compound Y27 about its structure and application. The authors work for Beijing Institute of Pharmacology & Toxicology.

## Authors' contributions

WXZ conceived the study, participated in its design and coordination and helped to draft the manuscript. YXZ also participated in the design of the study, performed the statistical analysis and drafted the manuscript. SHC and JPC carried out the animal experiments and immunoassays. RFY synthesized and provided compound Y27. YXZ and LHY have contributed ideas to the project. All authors read and approved the final manuscript.
